# Annual Meeting of the Medical Association of Georgia in Atlanta

**Published:** 1894-02

**Authors:** 


### Editorial.

### ANNUAL MEETING OF THE MEDICAL ASSOCIATION OF GEORGIA IN ATLANTA.

After making the circuit of the State this body comes back to the capital on the 18th, 19th, and 20th of April for its reunion. It is notable that the interest of members in the work of a scientific character has increased in later years and there is much less wire-pulling and political maneuvering than formerly. A few designing men at one time controlled matters in advance or in the course of the proceedings, but now all who care to participate in business affairs can have a voice in the deliberations, and there is no place for a small coterie or clique to exercise any undue influence over election of officers or in other business matters. The results are the legitimate expression of the majority of the members from the different sections of the State, representing the towns as well as the country places. It is therefore a greater inducement to attend the meetings and to take part in the proceedings. It is important that our country doctors shall realize that they are a part and parcel of the movement for the advancement of the profession. They are in many cases favorably situated for reflection upon the issues of treatment in diseases while making their trips to patients at a distance from each other, and thus reach conclusions which the active city practitioner fails to attain in his hasty calls upon a large number of patients. There is generally more original thought displayed by a country practitioner than by the city doctor, as the former must necessarily depend largely upon his own resources, whereas the latter can and does avail himself of a consultation when difficulties are presented in the management of a case of sickness or a surgical case. The country doctor should therefore bring his report of cases to our meetings and join in the discussion of matters pertaining to the treatment of patients, and every facility should be afforded for overcoming their reluctance to appear in public. They are able to contribute papers of general interest to the medical profession.

It devolves upon this Association to mould not only the views of medical men, but to exert its sway over the people outside of the profession. There is an urgent demand for education of all classes in regard to their welfare connected with the practice of medicine and surgery in their neighborhood, and for the elimination of quackery from every portion of our population. We should not rest satisfied with inculcating proper doctrines upon the medical profession, but we are also called upon to enlighten the people as to their true interests in the progress of our work. Unfortunately the self-laudation of charlatans finds a ready ear on the part of men and women who read their announcements, and to the chagrin of every high-minded medical man, there are members of the profession who do not scruple to follow their example by thrusting themselves before the public by so styled interviews and by news items of their attendance upon patients, which have no other end than to serve as advertisements.

It should be one of the objects of the State Association to put a stigma upon all this kind of trickery, whether in high or low places; and if the Judicial Council would bring every member who had been guilty of such pratices before its tribunal for strict enforcement of the ethical requirements, it would put a stop to such subterfuges. I admit that the motives of a member may sometimes be questioned in giving notice of the departure of a colleague from the path of rectitude, but if facts can be presented from the printed records, no one should hesitate to make such reports to the council at each meeting. It may be thrown in my teeth that all are more or less amenable to censure, and let him who is guiltless throw the first stone; yet there are degrees of guilt which shall indicate those offenders who are proper subjects of punishment to serve as a terror to evil-doers.

The Association made a move in the right direction at the last meeting by the appointment of a committee of one from each congressional district, for the presentation to the legislature of such matters as called for action by that body. The chairman will doubtless report at the forthcoming meeting the untoward result of the labors of that committee, due to a great extent to the opposition of two members of our profession who were in the House of Representatives. We should not abandon the work which has been undertaken on account of the miscarriage of the bills for an Examining Board and for a Board of Health in the last legislature. Renewed and more vigorous efforts should be made to carry out the measures inaugurated by the Association; and every medical man in the several sections of the State should make a personal appeal to his representative to vote for the passage of these or similar bills for the benefit of all concerned.

It is not expected that such enactments shall redound to the interest of the medical profession so much as to the benefit of the people generally; but on the same line that hygiene is advocated by medical men for the general good, these reforms should be presented by our profession in its corporate capacity as a State Association. If the population of the State of Georgia could be informed in regard to the evils which are sought to be remedied by a Medical Examining Board and a Board of Health in this State, there would be such an amount of influence brought to bear upon the members of the legislature as to secure a favorable consideration at the next session.

The propriety of presenting the data relative to these measures, in a concise form, to every voter in Georgia is worthy of consideration by the Medical Association at its forthcoming meeting; and it could be done without great expense in the form of a printed circular through the mail. J. M'F. G.

